# Optimal Design, Characterization and Preliminary Safety Evaluation of an Edible Orodispersible Formulation for Pediatric Tuberculosis Pharmacotherapy

**DOI:** 10.3390/ijms21165714

**Published:** 2020-08-10

**Authors:** Nyaradzo Matawo, Oluwatoyin A. Adeleke, James Wesley-Smith

**Affiliations:** 1 Division of Pharmaceutical Sciences, School of Pharmacy, Sefako Makgatho Health Science University, Pretoria 0208, South Africa; nmatawo1@gmail.com; 2 Electron Microscope Unit, Sefako Makgatho Health Science University, Pretoria 0208, South Africa; jaime.wesley-smith@smu.ac.za

**Keywords:** Orodispersible formulation, pyrazinamide, pediatric drug delivery, tuberculosis, design of experiments, children, edible films

## Abstract

The severity of tuberculosis (TB) in children is considered a global crisis compounded by the scarcity of pharmaceutical formulations suitable for pediatric use. The purpose of this study was to optimally develop and evaluate a pyrazinamide containing edible orodispersible film formulation potentially suitable for use in pediatrics actively infected with TB. The formulation was prepared employing aqueous-particulate blending and solvent casting methods facilitated by a high performance Box Behnken experimental design template. The optimized orodispersible formulation was mechanically robust, flexible, easy to handle, exhibited rapid disintegration with initial matrix collapse occurring under 60 s (0.58 ± 0.05 min ≡ 34.98 ± 3.00 s) and pyrazinamide release was controlled by anomalous diffusion coupled with matrix disintegration and erosion mechanisms. It was microporous in nature, light weight (57.5 ± 0.5 mg) with an average diameter of 10.5 mm and uniformly distributed pyrazinamide load of 101.13 ± 2.03 %*^w^*/*_w_*. The formulation was physicochemically stable with no evidence of destructive drug–excipient interactions founded on outcomes of characterization and environmental stability investigations. Preliminary inquiries revealed that the orodispersible formulation was cytobiocompatible, palatable and remained intact under specific storage conditions. Summarily, an edible pyrazinamide containing orodispersible film formulation was optimally designed to potentially improve TB pharmacotherapy in children, particularly the under 5 year olds.

## 1. Introduction

The latest World Health Organization’s (WHO) global report estimated that approximately 10 million people developed new tuberculosis (TB) infections that progressed to TB disease with about 1.5–2 million deaths recorded per annum [1]. So far, TB is the deadliest infectious disease globally and millions of people continue to fall sick and die annually. It is amongst the first ten primary causes of death from a single infectious agent worldwide, ranking above HIV/AIDS [1,2,3] (WHO, 2019; Swaminathan and Rekha, 2010; Kumar et al., 2017;). It remains a global threat with approximately 1.7 billion people having latent TB infection that can turn into active TB disease at any time [1]. Tuberculosis is an airborne, infectious disease that usually affects the lungs (pulmonary TB) leading to severe coughing, fever and chest pains or in some rare cases, other body parts (extra-pulmonary TB). It is caused by *Mycobacterium tuberculosis* also called tubercle bacilli. It is preventable and curable if diagnosed early and managed with the correct medicines [1,4,5]. 

Generally, TB infection within the pediatric population is considered to be a major cause of morbidity and mortality [6]. According to the latest WHO’s global TB report, at least one million children under the age of 15 (accounting for about 11% of the affected population) contract active TB infection with about 230,000 fatalities recorded annually [1] (WHO, 2019). Children may have TB disease at any age, but most often under 5 years old in TB-endemic countries. TB disease is also prevalent amongst children infected with the human immunodeficiency virus (HIV) who are usually at a twenty times greater risk of contracting active TB infection compared to children who are HIV negative [7,8]. Children often contract TB from actively infected adult household members, during birth or when they present with weak immune systems; such as in infants, those infected with HIV or the severely malnourished who are at greater risk of developing TB disease or even dying. Pulmonary TB is the most common in children although extra-pulmonary TB may occur. Pediatric TB is more common in developing countries where there is overcrowding, poverty and malnutrition than in developed states [2]. Treatment and prevention of TB in children is considered neglected regardless of the alarming statistics as there are few scientifically justified studies focusing on: (i) accurate pediatric dosing; (ii) designing desirable drug formulations suitable for use in children of all ages; (iii) developing effective diagnostic tools for this age group as they usually do not manifest any symptoms or signs of disease early; plus (iv) the belief that childhood TB is not important for TB control [9,10,11]. 

To date, commonly used pharmaceutical formulations are either liquid dosage forms (e.g., solutions, suspensions), fixed dose dispersible tablets and in most instances, adult tablets are often broken, crushed or mixed with food or water (co-administration) to make pediatric management possible [12,13,14,15]. Despite the availability of a few commercialized pediatric preparations, considerable global scarcity still exists, meaning that many children are unable to access these medicines [15,16,17,18,19]. Moreover, studies have shown that co-administration (with food, water etc.) is a common global practice for treating children with TB and that it is performed without appropriate instructions. In most case, caregivers just choose any food or drink without any assessment of its impact on safety and efficacy [13,15,20]. This may potentially lead to inaccurate dosing, resulting in reduced efficacy or adverse effects often caused by under-dosing and over-dosing respectively, disruption of the outer coating leading to physicochemical instabilities, and potential active pharmaceutical ingredient (API) wastage [2,13,21]. 

The use of alternative dosage forms such as suspensions or solutions can potentially help us overcome some of these challenges but they are also known to be generally less stable even when refrigerated, difficult to taste mask, expensive for safe transportation and have short shelf lives; all of which limit their applicability [2,22]. Dispersible tablets on the other end are deemed more child-friendly but still limited in that they are difficult to administer while in transit or when there is reduced/no access to potable water—like in most underdeveloped and developing countries where TB is endemic. They usually contain additives that are either not safe for use in children or hygroscopic in nature, making them prone to atmospheric moisture/water absorption that can lead to active drug instability, eventual inactivity and possible pharmacotherapeutic inefficacy [17,19,23]. Other potentially applicable delivery systems for children include chewable tablets, which are often more suitable for older children (>3 years) with teeth, and sprinkles, though they are more acceptable for older children that can eat solid food [23,24]. 

Recent studies show that the most popular age appropriate delivery systems are small sized, solid oral drug delivery systems e.g., minitablets and multi-particulates and orally disintegrating formulations like orodispersible tablets or films [18,25]. Particularly, orodispersible formulations are of choice because of their characteristic advantages such as water free administration, easy to use anywhere and at any time without the need for external help or specialized caregivers, improved stability, easier transportation, cost effectiveness and rapid disintegration when placed within the oral cavity releasing incorporated API for absorption. This definitely allows easy administration to pediatric patients with or without teeth [26]. Orodispersible delivery systems offer advantages such as enhanced pediatric compliance, possibility of local action, dosage accuracy, reduced choking risks, easy handling and portability [27,28]. They also allow rapid onset of action and increase in bioavailability due to rapid dispersion within the mouth and significant pre-gastric absorption, all leading to desirable pharmacotherapeutic efficacy [29]. Furthermore, antitubercular agents are administered at low doses in children so, orodispersible formulations will not be outsized or pose a choking hazard [19,23,24,25,30,31].

Therefore, this study details the design, optimization and systematic in vitro evaluation of a polymer-based, orodispersible film formulation containing pyrazinamide (PZA) as a potential alternative for flexible pediatric dosing. It is a first line antitubercular agent often used in combination with isoniazid, rifampicin and ethambutol for the treatment of active TB infection [32,33]. PZA is highly bactericidal, and acts by sterilizing slowly metabolizing tubercle bacilli, resulting in low incidence of bacteriological relapse post completion of chemotherapeutic regimen. It facilitates treatment shortening, leading to greater patient compliance [32,34,35,36,37,38,39]. It is a prodrug which undergoes conversion into active pyrazinoic acid by the bacterial enzyme pyrazinamidase at or below pH 5.6 [33]. Typically, it is administered for the initial 2 months of a 6-month treatment for drug-susceptible infections. PZA is a Class III drug according to the Biopharmaceutics Classification System (BCS) characterized by its high aqueous solubility (15 mg/mL at 25 °C), relatively low permeability (logP = −1.88) and linear absorption over a broad spectrum of doses [36,38] (Becker et al., 2008; Adeleke et al., 2016). The PZA loaded orodispersible matrices were prepared using the solvent casting method [27,40,41]. The PZA loaded formulation was prepared using a combination of pharmaceutical excipients which included copolymer polyvinyl alcohol-polyethylene glycol as a matrix and film forming agent, citric acid as a natural preservative, sodium starch glycolate as a superdisintegrant and xylitol as a sweetener acceptable for pediatric use as documented by Dixit and Puthli [42]. Formulation preparation and optimization were facilitated using a response surface method based on a 4-factor, 3-level Box Behnken experimental design (Minitab^®^ 18 Statistical Software (Minitab LLC, State College, PA, USA), a robust, high performance quadratic template widely applied in the development of viable drug carriers [38,43,44]. The optimized orodispersible film formulation was then physicochemically characterized in vitro by determining its mass, dimensions (inner and outer diameter), disintegration time, drug release and kinetics, drug content, dissolution pH, surface morphology changes, thermal behavior, crystallinity and structural chemical backbone transitioning. Furthermore, we studied the stability of the optimized formulation under common environmental storage conditions, its organoleptic qualities and cytobiocompatibility in vitro.

## 2. Results and Discussion 

### 2.1. Orodispersible Formulation Variants

Employing the initial one-variable-at-a-time screening together with the systematic 4-factor, 3-level Box Behnken experimental design template, 27 pyrazinamide loaded orodispersible formulations were successfully prepared using the solvent casting method. Through these approaches, the independent variables affecting the response parameters, namely disintegration time (Y_1_), dissolution pH (Y_2_) and formulation weight (Y_3_), were identified. In general, the orodispersible formulations appeared as whitish, dense and bendable, hollow cup-shaped matrices that were light weight (<122 mg) and had average inner and outer diameters of 11 ± 1 mm and 10 ± 0.81 mm respectively. Overall, the 27 orodispersible formulations had average weights ranging between 121.4 ± 8.00 mg and 60.87 ± 3.80 mg, disintegrated with a total matrix structure collapse within 0.20 ± 0.09 to 5.67 ± 0.42 minutes and presented dissolution pHs spanning from 6.59 ± 0.23 to 7.43 ± 0.01 which is relatively close to that of the oral cavity (saliva). Differences observed in the measured response parameters showed that the selected independent variables applied at the varying factors levels and combinations, according to the quadratic design template, had noteworthy effects on the nature of each formulation. Numerical values of response parameters measured for all 27 formulations are presented in Table 1.

### 2.2. Selection and Validation of the Optimized Orodispersible Formulation

The ANOVA analysis revealed that the percentage of polyvinyl alcohol polyethylene glycol, sodium starch glycolate, citric acid and xylitol contained in each formulation variant significantly (*p* < 0.05) impacted the response parameters. Based on the statistical method and constraints applied on the formulation weight, disintegration time and dissolution pH, a formula was developed for the preparation of the optimized orodispersible formulation using the Minitab^®^ 18 Statistical Software. An overall desirability of 0.991 indicating the robustness of the optimization platform and design template was obtained. The optimized formula containing the levels of each independent variable is shown in Table 2. To further confirm the validity and suitability of the optimization process, the optimized formulation was prepared in triplicate following earlier described method and measurements of the formulation weight, disintegration time and dissolution pH were performed experimentally. The magnitude of the observed response parameters measured against that of the predicted values displayed a high degree of correlation, further supporting the precision and robustness of the statistical design employed for generating the desired optimized formulation. 

### 2.3. Physical Properties of Optimized Formulation

**Figure 1:**
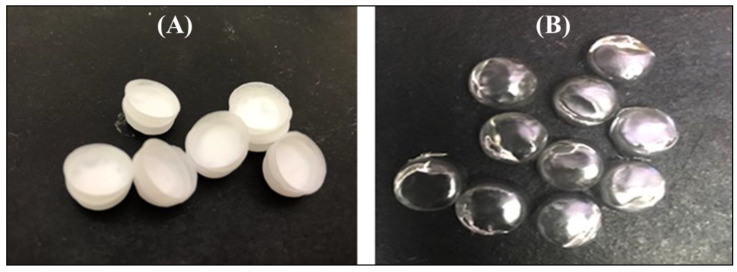
Digital photograph of (**A**) drug loaded and (**B**) placebo orodispersible film formulation.

The optimized orodispersible formulation was thin, flexible making handling possible, whitish in color with a hollow/concave shape as well as inner and outer diameters of 10.00 ± 0.52 mm and 11.00 ± 0.43 mm. The formulation was made up of sodium starch glycolate as a super disintegrate, co-polymer polyvinyl alcohol polyethylene glycol as a matrix and film forming agent, xylitol as a sweetener suitable for pediatrics, citric acid as a natural preservative and pyrazinamide (500 mg) as a model antitubercular agent. The optimized formulation was light weight and small enough for orodispersible applications. It rapidly disintegrated in less than 60 seconds (i.e., 0.58 min ≡ 34.98 s) when placed in simulated saliva at 37 ± 0.1 °C. The dissolution was close to neutral (7.0) and saliva pH (6.8), meaning that the formulation has no potential to irritate the buccal mucosa (Table 2). Digital photographs of the PZA loaded and drug free (placebo) optimized formulations are shown in Figure 1.

### 2.4. Drug Content and Release Behavior 

**Figure 2:**
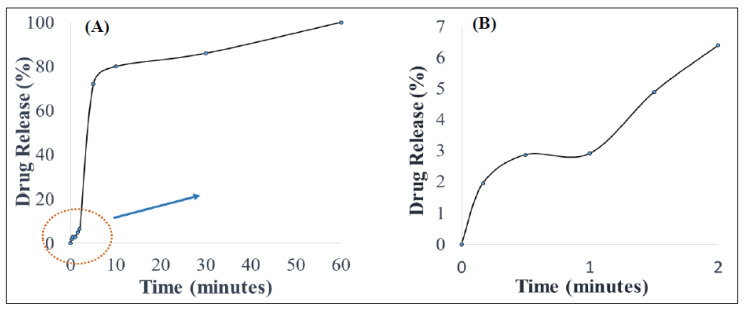
(**A**) In vitro drug release behavior in simulated saliva and (**B**) illustrates an expanded segment of the drug release profile.

The PZA content of the optimized orodispersible formulation was 25.02 ± 0.71 mg equaling 101.13 ± 2.03%*^w^*/*_w_*, indicating that uniform drug distribution occurred among replicate test samples and drug remained stable during and after preparation. In vitro drug release was carried out under biorelevant conditions to determine the rate at which PZA molecules were released in simulated saliva (pH 6.8) at 37 ± 0.1 °C, mimicking the buccal environment. The generated release profile is illustrated with Figure 2. Figure 2B, which is an expanded form of segments of Figure 2A, shows that drug liberation was initiated under 10 s (0.17 min) with 1.94 ± 0.28% released almost immediately after the formulation came in contact with the dissolution media (onset of matrix disintegration). Subsequently, the amount of drug released continued to increase rapidly, reaching its first peak at 5 min (72.01 ± 11.93%) and then maintained a relatively plateaued profile until 60 min when complete matrix dissolution and drug release (102.50 ± 5.19%) occurred. 

The mathematical models applied to represent the release mechanisms of the active drug from the formulation were zero-, first-, second-order as well as Higuchi, Korsmeyer–Peppas and Hixon–Crowell models yielded R values of 0.59, 0.47, 0.33, 0.40, 0.90 and 0.51, respectively. The optimized formulation displayed a good fit to the Korsmeyer–Peppas (*R = 0.90*) and the computed *n-value* was 0.83, meaning that drug release after formulation hydration was controlled by an anomalous drug diffusion process followed by matrix relaxation (disentangling polymer chains or disintegration) and erosion (matrix dissolution) [45].

### 2.5. Optimized Formulation Characterization 

#### 2.5.1. Thermal Behavior Using Differential Scanning Calorimetry and Thermogravimetry

**Figure 3:**
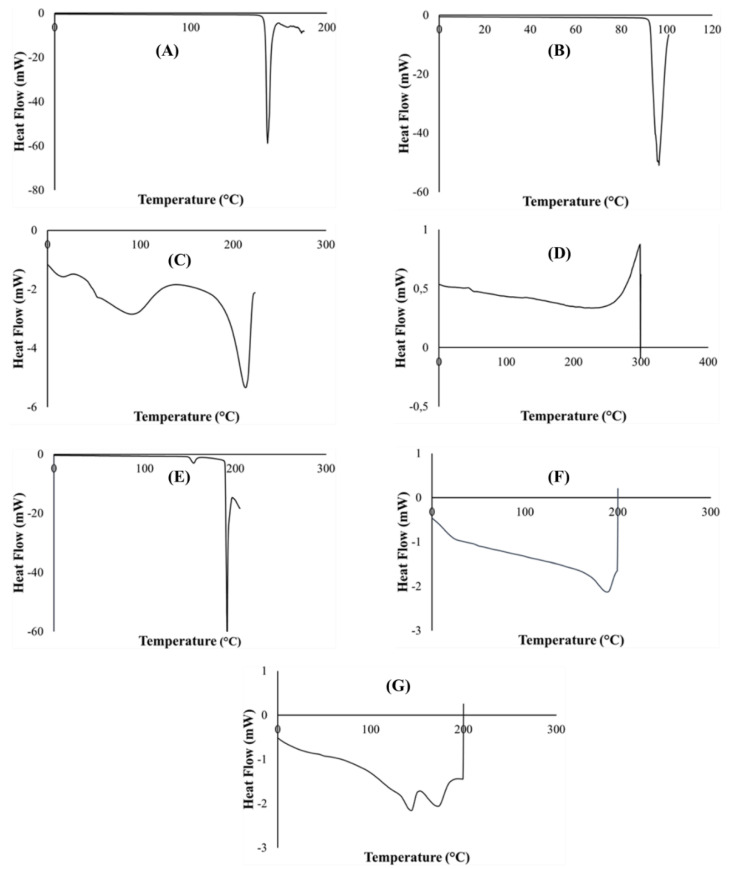
DSC Thermograms of (**A**) citric acid, (**B**) xylitol, (**C**) polyvinyl alcohol polyethylene glycol, (**D**) sodium starch glycolate, (**E**) pyrazinamide, (**F**) placebo and (**G**) drug loaded formulation.

Generated differential scanning calorimetry (DSC) thermograms were employed in the assessment of typifying thermal quantity changes for pure PZA, pharmaceutical excipients (citric acid, xylitol, poly vinyl alcohol polyethylene and sodium starch glycolate), optimized drug loaded and placebo formulations. Measured key thermal quantities include melting point (T_m_) and glass transition temperature (T_g_). Typical DSC thermograms are shown in Figure 3. The DSC scans of citric acid and xylitol represented in (Figure 3A,B) show sharp endothermic peaks corresponding to their T_m_ at 153.38 °C and 92.64 °C, respectively, indicating their purity and stable states. Polyvinyl alcohol polyethylene glycol (Kollicoat^®^ IR) thermogram (Figure 3C) depicts multiple broad endothermic and exothermic peaks with a small endothermic peak at 92.3 °C and a more prominent endothermic peak at 212.65 °C as T_m_. The presence of two endothermic peaks shows that the Kollicoat^®^ IR is made up of two different natured polymers. The appearance of Tg noted at 44.24 °C can be associated with its amorphous co-polymeric transitioning into a crystalline material. Sodium starch glycolate thermogram (Figure 3D) displayed characteristic exothermic peak at 298.75 °C and glass transition was observed at 46.45 °C. Pure pyrazinamide shows two endothermic peaks at 154.00 °C, corresponding to solid–solid transition and a sharp endothermic peak at 190.64 °C (Figure 3E), which corresponds to the T_m,_ indicating its α- polymorphic form and purity of PZA [38,46,47]. Thermal peaks identified for each excipient and pure drug are indicative of their purity and stability as individual compound before their inclusion in the orodispersible formulation mix. The placebo thermogram (Figure 3F) presented broad endothermic and exothermic peaks, revealing its semi-crystalline nature which can be related to its crystalline, semi-crystalline and amorphous constituents already mentioned above. Likewise, the optimized PZA loaded orodispersible formulation thermogram (Figure 3G) also displayed a semi-crystalline trend with broad endothermic and exothermic peaks occurring within the melting point region of excipients. This finding further supports its blended pure drug and excipient content. The slight shift of the PZA peak in the mixture may have been influenced by polymeric crystallization occurring during the formation of the orodispersible formulation resulting in the endothermic peak slightly shifting to 193 °C, accounting as the T_m_ of the newly prepared drug loaded formulation further confirming drug stability within the drug delivery matrix. The disappearance of melting peaks of individual excipients in the drug loaded formulation shows complete solubilization of the drug and excipients within the matrix. It also supports the formation of a new compound, hence the physical transitioning of PZA from crystalline to the amorphous form potentially accounting for the rapidly disintegrating quality of the developed formulation [48]. The obtained DSC thermograms demonstrate the compatibility of the drug (PZA) and pharmaceutical excipients used in the formulation development with no evidence of possible adverse interactions.

**Figure 4:**
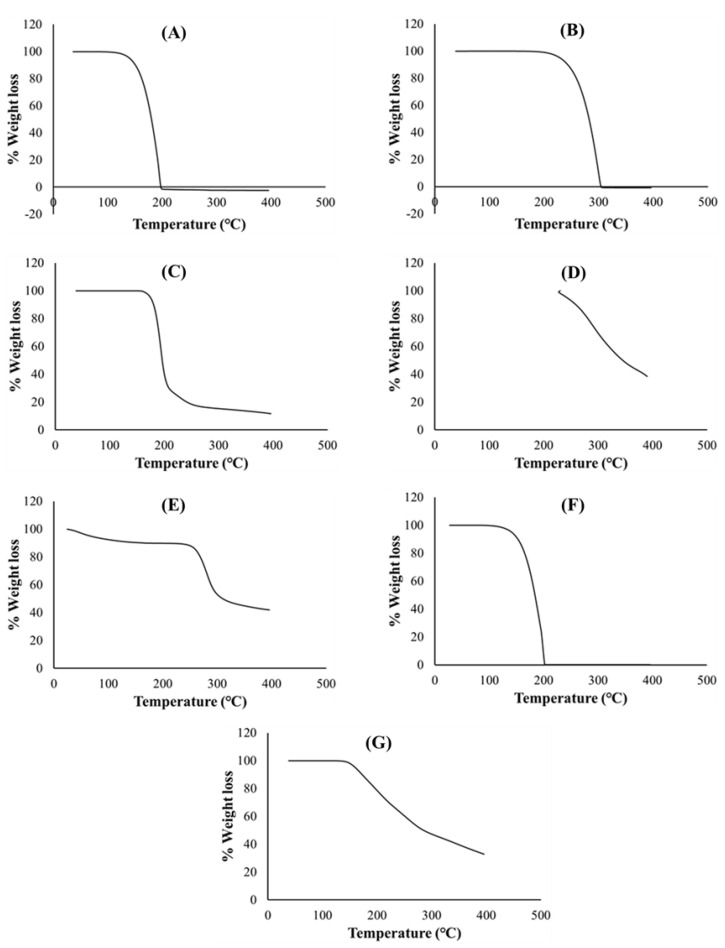
Thermogravimetric curves of (**A**) PZA, (**B**) xylitol, (**C**) citric acid, (**D**) polyvinyl alcohol polyethylene glycol, (**E**) sodium starch glycolate, (**F**) drug loaded formulation and (**G**) placebo.

**Figure 5:**
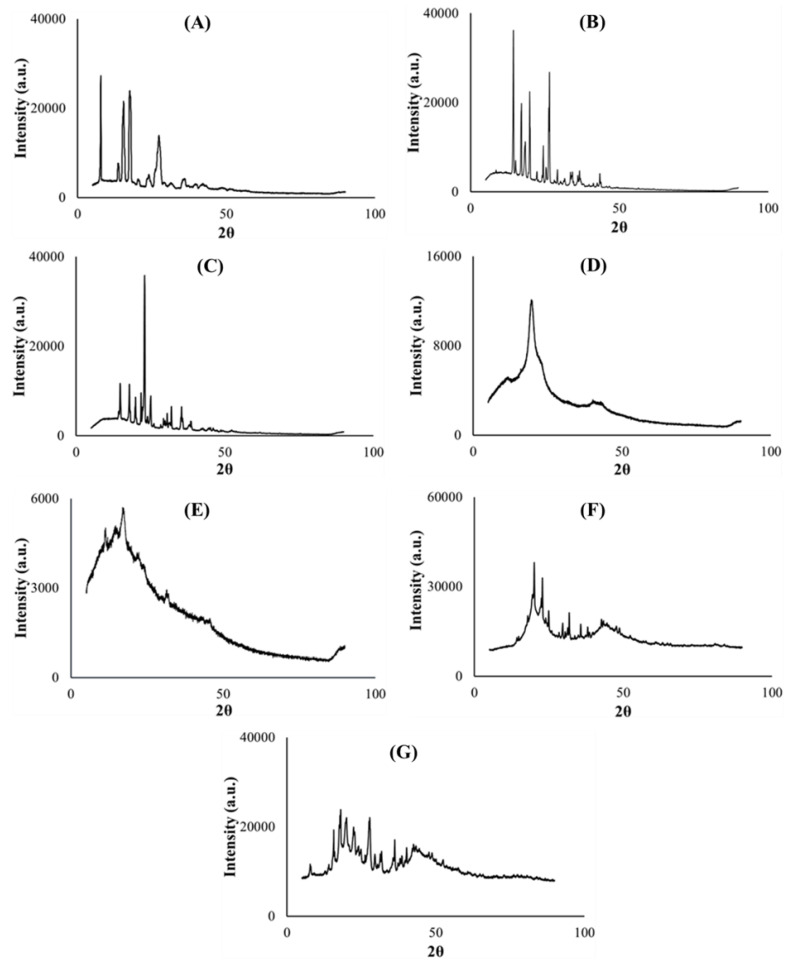
X-ray diffractograms of (**A**) PZA, (**B**) citric acid, (**C**) xylitol, (**D**) polyvinyl alcohol polyethylene glycol, (**E**) sodium starch glycolate, (**F**) placebo and (**G**) drug loaded formulation.

Thermogravimetric curves of pure PZA, pharmaceutical excipients, optimized drug loaded and placebo formulations recorded under a nitrogen saturated atmosphere, purge and heating rates of 25 mL/min and 10 °C/min, respectively, are presented in Figure 4. TGA analysis was conducted for additional investigation of thermal degradation events quantified as percentage weight loss as it relates to temperature and its impact of formulation stability. First, important thermal events were identified for individual excipients and pure PZA. The PZA thermal decomposition T_onset_ was observed at 164 °C and complete weight loss occurred at 199.15 °C (Figure 4A). The onset of thermal decomposition for xylitol (Figure 4B) was noted at 240.59 °C and final decomposition temperature was observed at about 306.73 °C, showing it thermal robustness as T_onset_ was above 200 °C. Citric acid and poly vinyl alcohol polyethylene thermal plots in (Figure 4C,D) commenced decomposition at a T_onset_ of about 185.99 and 236.39 °C, respectively, and complete breakdown only occurred at temperatures greater than 400 °C, confirming thermal stability of these excipients. Sodium starch glycolate (Figure 4E) showed two decomposition events, water loss from the polymer was observed at about 125.01 °C, corresponding to 8% weight loss, followed by final decay at 257.99 °C. Generally, the excipients presented with relatively high, single decomposition temperatures, 199 °C and above, indicating their distinct stability and purity. Thermal decomposition of optimized drug loaded and placebo (Figure 4F,G) began at 156.24 and 200.38 °C, respectively. Complete weight loss followed at 202.72 °C for the PZA loaded formulation and beyond 400 °C for the placebo. Onset temperatures considerably below 200 °C suggests lower thermal stability which, in this case, can be linked to the loss of residual water molecules from the both matrices due to the presence of hydrophilic components in both drug loaded and placebo formulations. Usually, weak drug and hydrophilic polymer interactions break easily around 100 °C [49]. The presence of an additional non-polymeric hydrophilic molecule, PZA, within the drug loaded formulation matrix probably accounts for the reduction in the onset temperature (156.24 °C) compared to that recorded for the placebo (200.38 °C) which contains all other hydrophilic constituent excluding PZA. This can further influence the temperature at which complete weight loss (100%) occurred for both drug loaded (202.72 °C) and placebo formulations (>400 °C). From these observations, it appears that the placebo contains more crystalline domains within its molecular structure and its components are more of a physical blend with less amorphous transitioning happening compared to the drug loaded formulation, where a degree of amorphization seems to occur between the PZA and excipient physical blend as also revealed through the DSC (Figure 3G) and XRD (Figure 5G) analytical outputs. Overall, the TGA thermograms exhibited the thermal stability of both drug and pharmaceutical excipients, either as separate entities or blends in the different formulations, as well as presented no visible trace of any destructive chemical interaction. Additionally, the similarity in weight loss patterns plus relative overlap in final decomposition temperatures for both drug loaded formulation (202.72 °C) and pure PZA (199.15 °C) (Figure 4A,F), can further signify formulation matrix stability and drug intactness which, was also the case with the DSC analyses as the PZA peak remained identifiable in the formulation blend with a slight shift associated with the presence of other excipients (Figure 3G).

#### 2.5.2. Crystallinity

Powder X-ray diffractometry was used to determine the physical, solid state changes and crystallinity [50] of the model drug PZA, pharmaceutical excipients, optimized drug loaded and placebo formulations. The diffractograms displayed in Figure 5A–C depict the crystalline nature of pyrazinamide, citric acid and xylitol correspondingly with distinct, high intensity peaks (>30,000 a.u.). Co-polymer polyvinyl alcohol polyethylene glycol is semi-crystalline (characteristic of it make-up), typified with broader band, less intense (<16,000 a.u.) crystalline peak observed at 19.2 (2*θ*) and multiple blunt regions (Figure 5D), while sodium starch glycolate exhibited a low intensity (<6000 a.u.), undefined topography (Figure 5E), which can be related to its amorphous character, largely contributing to the non-crystalline domains within the entire molecular structure of the optimized formulations. The optimized drug and placebo formulation diffractograms are considered a sum of individual diffractions generated by the component excipients and drug [26]. When compared with its drug loaded counterpart, the placebo diffractogram (Figure 5F) also exemplified the presence of recurrent amorphous and higher intensity crystalline regions (>30,000 a.u.) within its matrix which further validates findings documented from the TGA analysis where the placebo was shown to require higher temperatures to exhibit complete decomposition compared to the drug loaded formulation (Figure 4F,G). The optimized PZA formulation diffractogram (Figure 5G) irregularly exhibited slightly broader, sparse and lower intensity peaks (<30,000 a.u.) plus an expanse of blunt regions both associated with its crystalline and amorphous states respectively attributed to the excipients and PZA constituents. The presentation of the amorphous segments within the drug formulation structure appears substantial and may be linked with the rapid disintegration characteristic observed upon hydration, as the amorphous state is known to improve the solubility of drug delivery systems, thereby enhancing dissolution and drug absorption within the body [38,41,49]. The crystalline PZA appeared transformed into a partially amorphous state evidenced by the relative loss of its crystalline peaks intensity, particularly that identified at 8.03 (2*θ*) which was at about 30,000 a.u. and reduced to 10,000 a.u. in the optimized formulation diffractogram (Figure 5A). These findings also concur with the DSC and XRD analytical outputs. 

#### 2.5.3. Structural Interpretation

**Figure 6:**
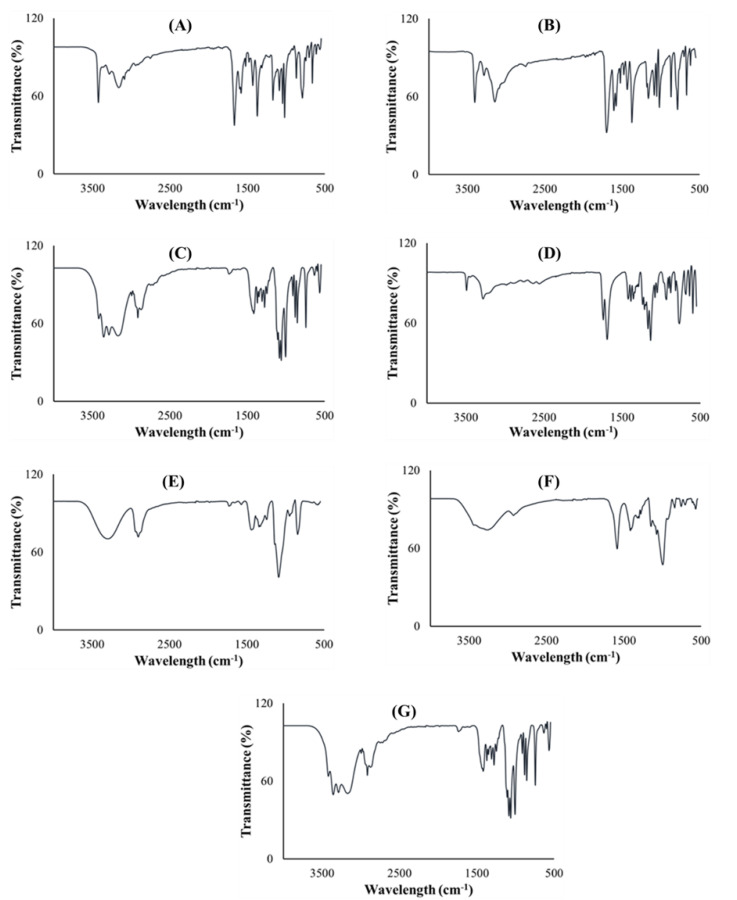
FTIR spectra of (**A**) xylitol, (**B**) sodium starch glycolate, (**C**) citric acid, (**D**) polyvinyl alcohol polyethylene glycol, (**E**) PZA, (**F**) drug loaded formulation and (**G**) placebo.

Fourier transform infrared spectroscopy was performed on all pure excipients, PZA, optimized drug loaded and placebo formulations to determine any drug–excipient interactions, and produced spectra are presented in Figure 6. The analysis was focused on identifying vibrational peaks that depict the presence of particular functional groups in the pure drug and pharmaceutical excipients (Figure 6A–G). Pinpointing xylitol peaks related to O-H stretching were observed at 3354 cm^−1^ and 3284 cm^−1^; an intense C-H peak at 1418 cm^−1^ (Figure 6A) while C-H and O-H peaks observed at 2916 cm^−1^ and 3438 cm^−1^, respectively, were specific for sodium starch glycolate xylitol peaks (Figure 6B). Typifying citric acid C-O-H peak was noted at 1372 cm^−1^, C-O vibration at 3282 cm^−1^ and O-H bending at 1172 cm^−1^ (Figure 6C). Specific peaks documented at 1090 cm^−1^, 2902 cm^−1^ and 3296 cm^−1^ correspond to C-O, C-H and O-H stretching, respectively; C-H bend at 1448 cm^−1^, C-H rock at 848 cm^−1^ and -C-C- vibration at 1086 cm^−1^ signify co-polymer polyvinyl alcohol polyethylene glycol—Kollicoat^®^ IR (Figure 6D) [51,52,53]. Of note are peaks representing N-H stretch at 3148cm^−1^, 3292 cm^−1^, 3388 cm^−1^ and 3408 cm^−1^, C-N (ring, stretching) at 1680 cm^−1^, C-C ring stretching at 1436 cm^−1^, C=N at 1162 cm^−1^, C=O at 786 cm^−1^ and C=C at 1704 cm^−1^, which are key to its structural make-up were logged for pure PZA (Figure 6E) [54]. The optimized PZA loaded formulation spectra (Figure 6F) displayed distinctive peaks, appearing within identical vibrational frequency range obtained for PZA but with slight shifts due to the presence of polymeric and non-polymeric additives. Peaks at N-H = 3290 cm^−1^, 3406 cm^−1^ and 3426 cm^−1^, C-O = 1680 cm^−1^ and 1022 cm^−1^, C-C (ring, stretching) = 1434 cm^−1^, typical of the standard PZA chemical backbone, were identified. A presentation of functional groups specific to the pure drug and/or excipients individually reflect in the generated spectra for the optimized drug formulation and exposes the level of structural compatibility amongst these components. This signifies that despite the relative amorphization noted for formulated PZA, typifying structural peaks remained noticeably unaltered, thereby demonstrating the absence of chemically disruptive interactions during the formulation development process. The placebo FTIR spectra (Figure 6G) showed C-O stretching peaks at 1064 and 1008 cm^−1^ including C-H stretching peak seen at 2916 cm^−1^ accounting for the presence of both poly vinyl alcohol polyethylene glycol and sodium starch glycolate, C-O-H peaks were detected at 1420 cm^−1^ and 1374 cm^−1^ representing citric acid and, vibrational frequencies at 3418 cm^−1^, 3382 cm^−1^ and 3282 cm^−1^ are characteristics of O-H peaks for associated with xylitol, polyvinyl alcohol polyethylene glycol and citric acid chemical backbone structures. These show that the placebo is a homogenous physical blend of the different excipients. Overall, the outcomes of the FTIR analysis indicated that the drug and excipients were well incorporated, compatible and stable with absence of any destructive intermolecular or intramolecular interactions.

#### 2.5.4. BET Surface Area and Porosity

**Figure 7:**
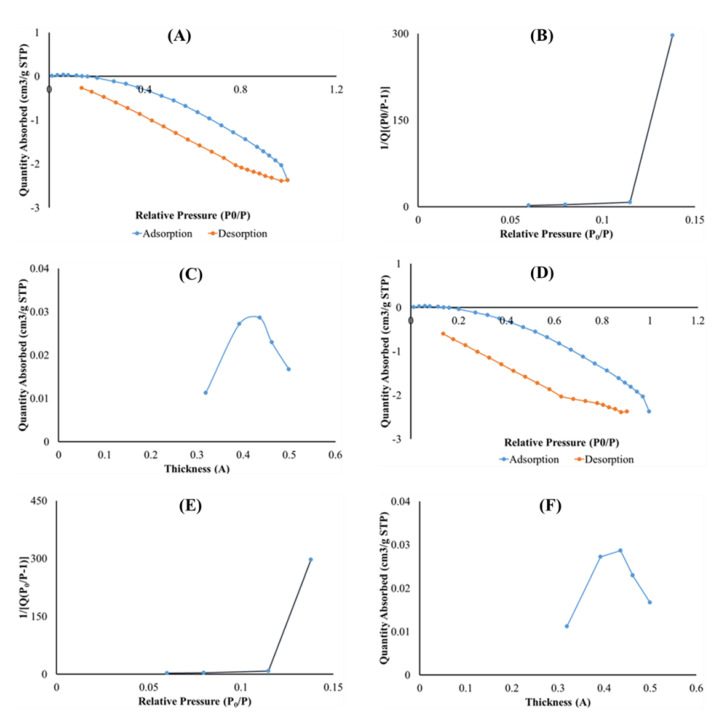
Graph presentation of Isotherm liner plot of (**A**) drug loaded and (**B**) placebo, BET surface area plot for (**C**) drug loaded and (**D**) placebo and t-plot of (**E**) optimized drug loaded and (**F**) placebo.

The surface areas of optimized drug loaded and placebo formulations were obtained by employing the theory of Brunauer–Emmett–Teller (BET) on nitrogen adsorption isotherms generated from the sample surface measured at 77 K. Typical isotherms are illustrated in Figure 7A,B, respectively. Drug loaded and placebo isotherms show similar trends at relative pressures of 0.2000–0.4000, revealing the existence of micropores in both formulations [55]. Specifically, the micropore volume of the placebo and drug loaded formulations were 0.00001 and 0.00003 cm^3^/g, respectively, and the presence of PZA within the matrix seems to increase the pore volume according to the numerical data. The specific BET surface area of drug loaded formulation (Figure 7C) and placebo (Figure 7D) were 0.0015 and 0.0753 m^2^/g, while the single point surface area measured 0.0020 and 0.1408 m^2^/g, respectively. The surface areas for the drug loaded sample is smaller than that of the placebo. This can be associated with the presence of successfully attached PZA molecules on the optimized formulation matrix. T-plots of the drug loaded (Figure 7D) and placebo (Figure 7E) profiles exhibited similar trends with the same thickness ranging between 0.3000 and 0.5000 nm, which may still be related to the finding that the PZA molecules are well incorporated into the formulation matrix, thus not changing its thickness either PZA-free or PZA-loaded. 

### 2.6. Typifying Surface Morphological Features and Transitioning with Hydration

**Figure 8:**
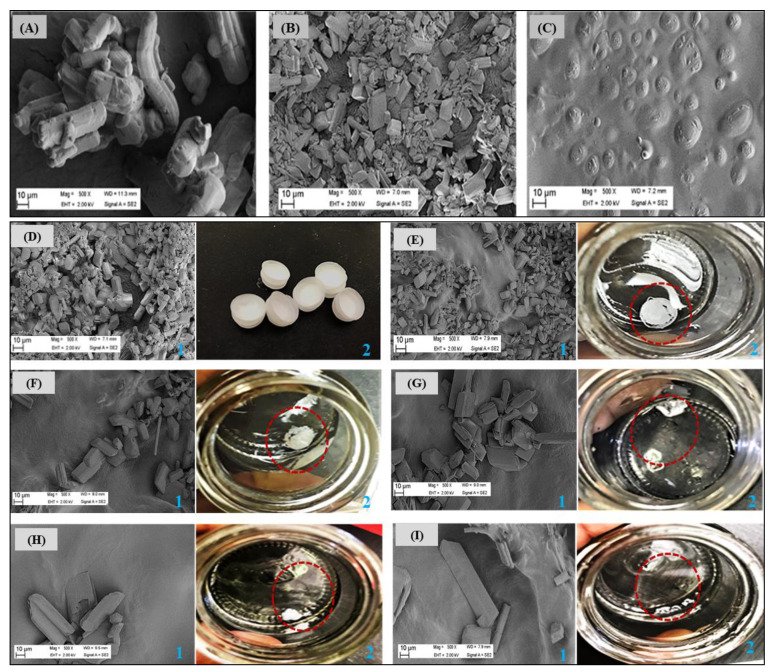
Scanning electron micrographs of (**A**) pure PZA, (**B**) optimized PZA loaded formulation, (**C**) placebo formulations. Changes are visible in the surface topographies of the unhydrated and hydrated optimized drug loaded formulation (**D**) before—0 s and after (**E**)10, (**F**) 30, (**G**) 60, (**H**) 90 and (**I**) 120 s of being in contact with simulated saliva under biorelevant conditions (pH 6.8; 37 ± 0.1 °C). Images were captured as SEM micrographs at magnification 500× (refer to the blue label “1”) and high resolution photographs (refer to blue label “2”) at each time point. The circular, red dotted lines shown in the photograph images (labelled “2”) represent left over formulation fragments after hydration.

Scanning electron microscopy was used to examine the differences in the surface morphologies of the anhydrous PZA, optimized drug loaded and placebo formulations as shown in Figure 8A–C, respectively. The pure PZA micrograph (Figure 8A) displayed irregular sized, rod shaped particles with defined edges confirming the crystalline nature of the PZA molecules [47,50]. The optimized PZA formulation surface micrograph (Figure 8B) revealed a uniform distribution of the drug molecules embedded throughout the carrier matrix, whereas the placebo (Figure 8C) showed a relatively plane topography with undulating segments confirming the absence of PZA molecules. 

Furthermore, time-dependent disintegration patterns of the optimized drug loaded formulation in preheated simulated saliva (pH 6.8; 37 ± 0.1 °C) was investigated using scanning electron microscopy and digital photography. Briefly, images were taken at 0 s (before hydration) and 10, 30, 60, 90 and 120 s after placement in simulated saliva. Captured micrographs and photographs showing changes in formulation surface morphology as it collapsed upon hydration and subsequently released PZA molecules over time are illustrated side-by-side (labelled 1 and 2) in Figure 8D–I. At time 0 s before hydration (Figure 8D), formulation matrix morphology was intact and displayed a wide coverage with PZA molecules (defined edge particles as per micrograph) as also discussed above. As formulation wetting progressed with time, gradual matrix collapse and breakdown occurred, and progressive migration of embedded PZA molecules ensued as a result of matrix disintegration (Figure 8D–I). Initial matrix collapse and drug molecule (visible as embedded particles with defined edges) migration into simulated saliva started at about 10 s (Figure 8E) and was more pronounced at 30 s (Figure 8F), followed by visible matrix erosion, dissolution and continuous outward movement of incorporated drug molecules as time elapsed (Figure 8G–I). These outcomes agree with the low disintegration time (34.98 s) recorded for the drug loaded formulation and is considered an indication of the desired rapid matrix fragmentation, which makes it an attractive and potentially suitable orodispersible delivery system for use in children. In essence, we were able to use the information provided from visualizing microscopic disintegration processes to validate macroscopic level formulation breakdown events.

### 2.7. Organoleptic Properties of Optimized Drug Loaded Formulation

Preliminary evaluation of organoleptic properties was based on color, texture (general appearance) and acceptability. Results showed that the orodispersible formulations placed on the tongue dispersed quickly—under 60 s in the presence of saliva (required no water for swallowing) and produced a generally satisfying taste as described by the panel. On the average, the volunteers rated the formulation 3.5, implying that the remaining bitterness was minor and mostly considered adequately taste masked/tasteless by them. The panelists also described the formulation color as acceptable, texture as satisfactory and easy to handle. Outcomes of this preliminary qualitative investigation makes the developed orodispersible preparation potentially attractive for pediatric use.

### 2.8. Cytobiocompatibility Evaluation

**Figure 9:**
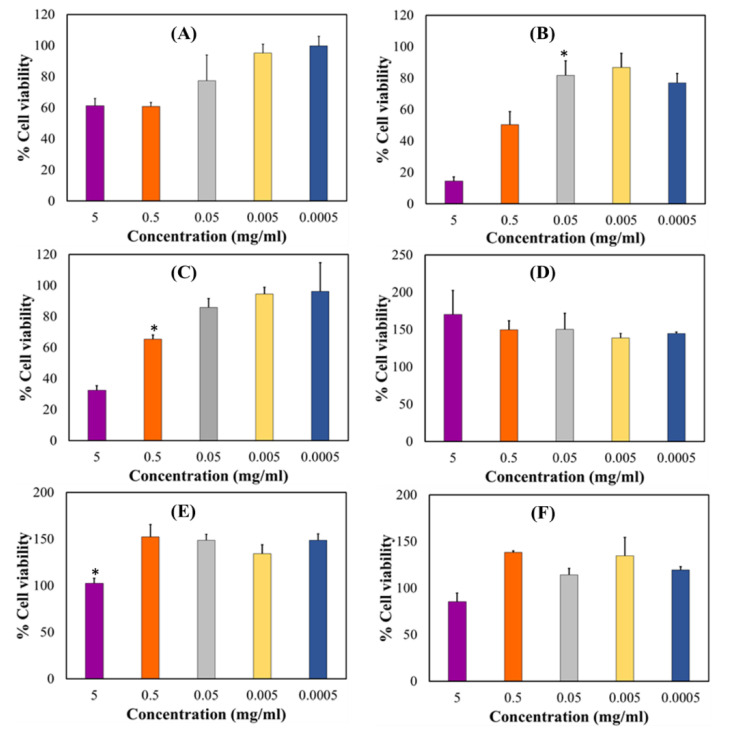
HepG2 cell biocompatibility levels measured by MTT analysis (**A**) drug loaded formulation, (**B**) placebo and (**C**) pyrazinamide and, neutral red assay (**D**) drug loaded formulation, (**E**) placebo and (**F**) pyrazinamide. Results represent mean ± SEM and statistical significance (*p* < 0.05) indicated with an asterisk (*)**.**

In vitro cytotoxicity assay was performed to determine the potential biocompatibility of the optimized drug loaded orodispersible formulation relative to the placebo and pure PZA using hepatocyte cell line (Hep2G) as a model. HepG2 cell line is commonly used to examine the toxicity of antitubercular drugs and respective formulations [56,57,58]. Cell viability was measured with the 3-(4,5-dimethylthiazol-2-yl) 2,5-diphenyl tetrazolium bromide (MTT) and neutral red (NR) assays at different xenobiotic concentrations ranging from 0.0005—5 mg/mL over a treatment period of 24 h. Cytotoxicity levels were presented as mean percentage cell viability and standard error of mean (SEM) for both MTT and NR assays (Figure 9). For the MTT assay, it was observed that cell viability generally increased as sample concentration decreased (Figure 9A–C). The optimized drug loaded preparation (Figure 9A) showed cell viability 60% and higher for all test concentrations while the placebo formulation displayed lowest cell viability <20% at 5 mg/mL, which consistently increased between 0.5 and 0.005 mg/mL and then insignificantly dropped to 77.09% at the lowest concentration of 0.0005 mg/mL (Figure 9B). Pure pyrazinamide, on the other hand (Figure 9C), expressed highest cell proliferation at lowest concentration 0.0005 mg/mL (96.29%) and the reverse at 5 mg/mL (32.40%). A combination of PZA and excipients in the formulation seemed to promote biocompatibility and cell growth in comparison to outputs captured for the placebo and PZA only. Interestingly, the neutral red analyses showed no cytotoxicity for all three test samples with values majorly greater than 100% for all concentration levels (Figure 9D–F). The only slight decrease in cell viability was noted for PZA at 5 mg/mL and it was statically insignificant. Hence, the NR uptake assessment revealed that drug formulation, placebo and PZA supported cell division and growth, an indication of cytobiocompatibility. These findings may be as a result of the differences in the biochemical reactions of both assays. The MTT assay is based on cellular respiration or mitochondrion cell metabolic activities while the NR analysis measures dye uptake and concentration within the lysosomes thus measuring staining capacity of live cells [3,59]. Consequently, a reduction in MTT-based cell viability can represent a decrease in metabolic activity. The fact that similar trends are not identified for the NR assay at all test concentrations, the cells can be considered to exhibit cytostatic effects at some point. This may mean that the introduction of the test compounds may have inhibited cell growth but does not necessarily promote cell destruction or death—as it is with the case of cytotoxicity. Identified cellular responses resulting from cell exposure to test samples can be termed as dose-dependent and two-phased, an occurrence related with hormesis which, is a two-way adaptive reaction of cells (biological systems) to external stress such as xenobiotics, environmental changes (e.g., pH, temperature) [60]. For both implemented assays, the optimized PZA orodispersible formulation demonstrated no significant reduction in cell viability.

### 2.9. Drug Formulation Stability under Changing Storage Conditions

Pyrazinamide formulation stability under varying environmental storage conditions was evaluated over 12 weeks. Briefly, drug formulations were placed in airtight, glass jars containing desiccant bags and stored in: (a) a dark enclosure (23 ± 3 °C/65 ± 5% RH), (b) refrigerator (4 ± 2 °C) and (c) under regular room conditions (24 ± 3 °C/70 ± 5% RH). Tests were performed in triplicate per storage condition and the stability indicators quantified were inner and outer diameters, disintegration time, dissolution pH, weight, and drug content using earlier described methods. Results were reported as average ± standard deviation. Freshly prepared control formulations were tested immediately (time = 0 weeks) and stability indicators recorded in three replicates. At the 12-week time-point, samples stored in dark enclosures (23 ± 3 °C/65 ± 5% RH) and refrigerator (4 ± 2 °C) retained their physical shape, color and showed minimal variation in stability indicators compared to values recorded at the starting point. Samples stored under regular room conditions (24 ± 3 °C/70 ± 5% RH) with fluctuating light exposure were considerably unstable, evidenced by their physical discoloration and documented stability indicators relative to the control samples (Table 3). Summarily, the suggested storage conditions for the optimized PZA containing orodispersible pharmaceutical formulation would be in airtight vessels containing desiccant bags kept away from direct or fluctuating light sources and under ambient or refrigerator settings.

## 3. Materials and Method

### 3.1. Materials

Pyrazinamide, citric acid, sodium starch glycolate (Primojel^®^), xylitol, Dulbecco’s modified Eagle’s medium (DMEM), fetal bovine serum (FBS), L-glutamine, non-essential amino acids, penicillin/streptomycin, disodium hydrogen phosphate, potassium dihydrogen phosphate, sodium chloride, 3-(4,5-dimethylthiazol-2-yl) 2,5-diphenyl tetrazolium bromide (MTT) and neutral red (NR) cell viability assay were purchased from Sigma Aldrich (St. Louis, MO, USA). Copolymer polyvinyl alcohol-polyethylene glycol (Kollicoat^®^ IR) was procured from BASF (Ludwigshafen, Germany). Hepatocyte cell line (HepG2) was purchased from American Tissue Culture Collection (ATCC) (Manassas, VA, USA). All other chemicals employed were of analytical grade and used as received. 

### 3.2. Experimental Design 

#### 3.2.1. Constructing the Box Behnken Design Template

The systematic preparation and optimization of the PZA loaded formulation was based on a 4-factor, 3-level Box Behnken experimental design template, a response surface methodology (RSM), constructed utilizing the Minitab^®^ 18 Statistical Software (Minitab LLC, State College, PA, USA). The independent variables were the formulation excipients namely polyvinyl alcohol polyethylene glycol (X_1_), sodium starch glycolate (X_2_), citric acid (X_3_), xylitol (X_4_). 3-levels of the independent variables referred to as lower (−1), midpoint (0) and upper (+1) limits were selected for the construction of the design template as represented in Table 1. The dependent variables or responses were parameters key to the performance of the formulation and these included disintegration time (Y_1_), dissolution pH (Y_2_) and formulation weight (Y_3_). Factor level selection for each excipient was set on their ability to produce stable orodispersible formulations, which was based upon the one-variable-at-a-time approach (OVAT) [38,61]. The OVAT approach was implemented by changing one variable per time while keeping the others constant so as to determine the influence exhibited by each excipient. Accordingly, the Box Behnken design template generated 27 possible combinations (F1–F27) with 3 replicates at central points to minimize errors as presented in Table 4 [62]. Model estimation and significance level were executed using the analysis of variance (ANOVA) where *p*-values below 0.05 indicated statistical significance and correlation coefficient (R) closest to one (>0.9) was selected because of complexities associated with quadratic experimental design templates (Table 3).

#### 3.2.2. Preparation of Orodispersible Formulations

Pyrazinamide loaded orodispersible formulations were prepared using the solvent casting technique [27,40,41]. Each formulation consisted of different amounts of polyvinyl alcohol polyethylene glycol, sodium starch glycolate, citric acid and xylitol based on the design template detailed in Table 5. As a result, 27 orodispersible formulations were prepared with each, containing a fixed quantity of pyrazinamide which equaled 500 mg per formulation. Briefly, for every orodispersible formulation variants, all excipients (factor levels) and drug were carefully weighed on a calibrated analytical balance (AS220.R2 Radwag Wagi Electroniczone, Radwag, North Miami Beach, FL, USA) and added to 20 mL deionized water under continuous stirring (Digital Hotplate Stirrer, Model H3760-HSE; Lasec; Ndabeni, Cape Town, South Africa) at 500 rpm over 60 min at 37 ± 0.1 °C until a homogeneous slurry was formed. The homogeneous mixture was left to cure in an airtight and dark environment until all air bubbles were visibly absent. Next, specific amounts (required to produce 20 films per formulation variant) of the cured slurry was filled into specialized, hollow plastic molds and then placed into a Labcon forced air circulation incubator (Model FSIH4, Krugersdorp, Gauteng, South Africa) until dried to constant weight at 25 ± 0.5 °C over 24 h. The resulting drug loaded formulations were then appropriately stored away in airtight, opaque vials for further testing. 

#### 3.2.3. Weight Determination for the Matrices 

Each prepared orodispersible formulation (F1–F27; Table 2) was weighed using a calibrated analytical balance (AS220.R2; Radwag Wagi Electroniczone, Radwag, North Miami Beach, FL, USA). For each measurement, three independent samples were weighed and mean weight ± standard deviation was calculated and recorded.

#### 3.2.4. In Vitro Disintegration Time and Dissolution pH of the Matrices

The in vitro disintegration time of the 27 experimental design orodispersible formulations was measured utilizing a modified petri dish method [63,64]. Disintegration time represents the specific period when formulation matrix collapse begins [38]. The disintegration time was determined visually using a dual-display digital stopwatch (Fotronic Corporation, Melrose, MA, USA). In this case, each sample was placed in 5 mL of pH 6.8 simulated saliva solution contained in a glass vial and placed in the shaking water bath (ST 30, NÜVE, Akyurt, Ankara, Turkey) maintained at 37 ± 0.1 °C and 10 rpm to mimic the oral cavity [26]. The vial was swirled after every 10 seconds and physical appearance of the formulation was consistently observed for any dimensional changes [28]. The simulated saliva was prepared by dissolving 2.38 g disodium hydrogen phosphate, 0.19 g potassium dihydrogen phosphate and 8.00 g sodium chloride in a liter of distilled water [65]. In vitro disintegration time was recorded at the point when the sample started breaking apart. Thereafter, test samples were allowed to dissolve completely to form a homogenous solution and dissolution pH recorded using a pH meter (GmbH 8603, Mettler Toledo, Sonnenbergstrasse, Schwerzenbach, Switzerland) [40,66]. All the measurements were done in three replicates.

### 3.3. Formulation Optimization

The main objective of the statistical design approach was to develop an optimal pyrazinamide loaded orodispersible formulation. After generating a full quadratic polynomial regression which connected dependent with independent variables from the Box-Behnken design template, experimental outputs were fitted within set limits for predicting the optimal orodispersible formulation. Selection and analyses of optimized levels were performed using the Minitab^®^ 18 statistical software by simultaneously applying specific constraints on the dependent variables namely, disintegration time, dissolution pH and formulation weight, as presented in Table 6. Accuracy and efficiency of the statistical optimization process was measured using the desirability function in which case a value closest to one is indicative of precision. To validate the experimental design approach, the optimized orodispersible formulation was prepared in triplicate, dependent variables measured and obtained values were compared to the predicted values. Thereafter, more optimized drug loaded and placebo formulations were prepared for additional in vitro characterization and testing.

### 3.4. Physical Properties of the Optimized Orodispersible Formulation

#### 3.4.1. Weight Determination

The optimized formulation weight was measured in triplicate using a calibrated analytical balance as previously described.

#### 3.4.2. Measurement of Inner and Outer Diameter

The inner and outer diameter of the optimized formulation was manually measured in triplicate using a centimeter calibrated precision ruler. 

#### 3.4.3. Disintegration Time and Dissolution pH

The time elapsed at the onset of in vitro disintegration and the media pH after complete formulation dissolution was quantified using methods already detailed above.

### 3.5. Drug Content Analysis

Pyrazinamide loaded and placebo optimized formulations of about 12 × 10 mm dimension were separately dissolved in 100 mL of simulated saliva contained in an Erlenmeyer flask. The resulting aqueous mixture was placed on a digital hotplate magnetic stirrer (Model H3760-HSE; Lasec; Ndabeni, Cape Town, South Africa) set at 37 ± 0.1 °C and 500 rpm. The samples were visually monitored until a complete clear solution was formed. Subsequently, 1 mL of the clear solution was appropriately diluted in simulated saliva and passed through the 0.45 μm nylon syringe filter (Whatman^®^, GD/X syringe filters, Sigma Aldrich, Johannesburg, South Africa). The placebo formulation was also subjected to the same dilution and filtration processes as the drug loaded samples and used as blank measurements to nullify background absorbance associated with included excipients. Filtrates collected from both drug loaded and placebo samples were then separately analyzed by measuring absorbance using a UV/VIS spectrophotometer (Nanocolour^®^ UV/VIS, Macherey Nagel, Separations, Bellville, Cape Town, South Africa) set at a λ_max_ of 268 nm, specific for PZA [26]. The final absorbance measurements obtained from this differential computation were fitted into a linear calibration curve (*y = 654.34 x; R^2^ = 0.96*) to obtain the actual and percentage PZA content of the optimized formulation. All quantifications were performed using three replicate samples.

### 3.6. Evaluation of In Vitro Drug Release Kinetics

The in vitro drug release experiment was carried out on three separate optimized formulations. Each sample was separately enclosed in lidded glass vials containing 5 mL simulated saliva and the entire contrivance was immersed into a shaking water bath at 37 ± 0.1 °C under gentle agitation of 10 rpm, mimicking the buccal environment. Thereafter, 2 mL sample was collected and replaced with an equal volume of freshly prepared, temperature equilibrated simulated saliva (37 ± 0.1 °C) at different time intervals (10, 30, 60, 90 s and 2, 5, 10, 30, 60 min). The samples were then diluted, filtered using 0.45 μm Whatman^®^ nylon syringe and analyzed with a Nanocolour^®^ UV/VIS spectrophotometer at λ_max_ = 268 nm to detect drug absorbance which was eventually translated into percentage drug release values employing a linear polynomial equation (*y = 654.34 x; R^2^ = 0.96*). Furthermore, obtained drug release profile was analyzed employing model dependent methods namely zero-, first-, second-order as well as Higuchi and Korsmeyer–Peppas and Hixon–Crowell [67]. The model of best-fit optimally describing the mechanism of drug release from the optimized orodispersible formulation was selected based on the coefficient of determination (R^2^) closest to one. All mathematical fitting was performed using the KinetDS, version 3.0 open source software.

### 3.7. Physicochemical Characterization 

#### 3.7.1. Differential Scanning Calorimetry (DSC)

The thermal properties of PZA, all excipients used, optimized drug loaded and placebo were evaluated and compared using a differential scanning calorimeter (DSC, Q2000 DSC, TA Instruments, New Castle, DE, USA). Approximately 6 mg of each sample was placed into a flat bottomed standardized aluminum pan which was directly transferred into the calorimeter for testing purposes. For referencing, an empty aluminum pan was included for each measurement as needed. All test samples were analyzed three times at 10 °C/min^−1^, temperature range between −65 °C and 300 °C under an inert nitrogen gas flow rate of 25 mL/min. The thermograms obtained were recorded and analyzed.

#### 3.7.2. Thermogravimetric Analysis (TGA)

The drug model PZA, excipients, optimized drug loaded and placebo formulations were assessed using a thermogravimetric analyzer (TGA Q500 V20.13 Build 39, TA Instruments, USA). About 8 mg of each sample was separately placed into platinum pans, heated at a temperature range of 10–400 °C, flow rate of 5 °C/min and maintained under constant nitrogen and air flow set at 40 mL/min and 60 mL/min respectively. The percentage weight loss during each heating cycle was recorded using the TGA universal analysis software. Measurements were completed in triplicate and results expressed as the mean of the three readings.

#### 3.7.3. Evaluation of Structural Transitions 

A Fourier transform infrared (FTIR) spectrophotometer (Perkin Elmer Spectrum 100 Series, Beaconsfield, UK) equipped with the Spectrum V 6.2.0 software was utilized for the characterization of PZA, all excipients, optimized drug loaded and placebo formulation samples. The FTIR spectra of each sample were recorded in the transmission mode at a frequency range of 550–4000 cm^−1^. Each spectrum was an average of 32 scans combined in order to achieve a satisfactory signal-to-noise ratio. In all cases, spectra resolution was maintained at 8 cm^−1^ and the gauge force at 150. The compatibility of the samples was checked and FTIR spectra documented for further analysis. 

#### 3.7.4. Surface Area and Porosity Analyses

The surface area and porosity of optimized drug loaded and placebo formulations were quantified utilizing the Brunauer–Emmett–Teller (BET) analyzer (Micromeritics TRISTAR II 3020, Micromeritics, Norcross, GA, USA) employing nitrogen adsorption mechanisms. About 0.3 g of each sample was degassed under a vacuum environment overnight at 40 °C. The specific surface area for each specimen was calculated using the BET method with experimental points fixed at a relative pressure of 0.01–1. 

#### 3.7.5. X-ray Diffraction (XRD)

The differences in the crystalline structures of PZA, excipient, drug loaded and placebo formulations were identified using an X’Pert Pro Powder X-ray diffractometer (PANalytical, Westborough, MA, USA). Anode material used was copper based, machine divergence slit was set at 0.38 mm and measurements were performed using a reflection-transmission spinner. Measurement operations were carried out using 1.54 Cu K-alpha (1 and 2) radiation, 45 kV generator voltage and 40 mA tube current. Continuous scanning was performed at 0.026 scan step size and 126.99 s/step between 5° and 90° (2θ). 

### 3.8. Surface Conformational Transitions of Dry and Hydrated Formulations

First, the surface morphology of pure PZA, drug loaded and placebo formulations were viewed using the Zeiss Supra 55 SM Scanning Electron Microscope (SEM) (Carl Zeiss, Germany) at a 2 kV accelerating voltage. The samples were cut into small pieces, mounted on aluminum stubs using double sided adhesive carbon tape and then sputter coated with approximately 15 nm chromium using a Quorum T150 ES coater (East Sussex, UK) before imaging. 

Afterwards, the changes in the surface geometry of the optimized drug loaded formulation upon hydration under biorelevant conditions, similar to that earlier described for the disintegration analysis, were studied to further corroborate previously observed disintegration and drug release patterns. At predetermined time intervals (10, 30, 60, 90, 120 s), photographs of the observed physical changes were captured, then remnants of the disintegrating formulation were carefully collected and dried to constant weight with a Labcon forced air circulation incubator at 25 ± 0.5 °C. Subsequently, dried remnants collected at the different time points were processed as described above and mounted for viewing on a Zeiss Supra 55 SM Scanning Electron Microscope. Photomicrographs for both dry (whole) and hydrated samples were taken at 500× magnification.

### 3.9. Preliminary Organoleptic Evaluation

A single blinded approach was used to evaluate taste acceptability and physical appearance of optimized drug loaded orodispersible formulation (each containing 25 mg PZA) by human volunteers (*n* = 5) [68,69]. Each volunteer was requested to allow formulations disperse in their mouths and to record the taste of each formulation on the provided chart after some seconds (under a minute) before removing formulation remnants from their mouths without ingestion. All panelists were provided with potable water to thoroughly rinse their mouths of any formulation residue using drinkable water before evaluating another sample (each panelist assessed 3 samples). The bitterness and quality attributes were evaluated using a 4-point hedonic scale with 1 point = very bitter, 2 points = moderate to bitter, 3 points = slightly bitter and 4 points = tasteless/taste masked). An average numerical value indicating the overall acceptability of the formulation was computed [68,69].

### 3.10. In Vitro Cytotoxicity Assay

The PZA, PZA loaded and placebo optimized formulation were employed as samples for investigating the cytobiocompatibility using Hepatocyte cell line (Hep G2 also referred to as ATCC^®^ HB-8065™) was obtained from the American Type Culture Collection (Manassas, VA, USA). Two colorimetric assays were employed to quantify cell viability of the samples namely 3-(4,5-dimethylthiazol-2-yl) 2,5-diphenyl tetrazolium bromide (MTT) and neutral red (NR) cell viability assay.

#### 3.10.1. Cell Culturing and Sample Preparation

The hepatocyte cell line was cultured in Dulbecco’s modified Eagle’s medium (DMEM), 10% fetal bovine serum (FBS), 1% L-glutamine, 1% non-essential amino acids (NEAA), and 1% penicillin/streptomycin. Tissue culture flasks (75 cm^2^) were used to grow the cell in an incubator maintained at 37 °C in 5% of carbon dioxide. The cells were harvested and passaged when they were confluent. Assay samples were dissolved in Dulbecco’s modified Eagle’s medium (DMEM) with serial dilutions (5, 0.5, 0.005, 0.0005 mg/mL) and prepared samples evaluated using MTT and NR assay detailed below.

#### 3.10.2. MTT Cell Viability Assay

A modified technique outlined by Mosmann (1983) and Vistica (1991) was used for MTT viability assay [57,70]. HepG2 cells were seeded at a density of 40,000 cells/mL in a 96-well plate. The cells were left to be attached overnight, then they were exposed at different concentrations (μg/mL) of the samples. The spent medium was aspirated after 24 h of incubation at 37 °C and substituted in simple DMEM by a 0.5 mg/mL MTT. After another 3 h of incubation at 37 °C, the medium was removed and 200 μL DMSO dissolved the purple formazan crystals. A microplate reader (SpectraMax^®^ Paradigm^®^ Multi-Mode Detection Platform, Molecular Devices LLC, San Jose, CA, USA) measured the absorbance at 540 nm.

#### 3.10.3. Neutral Red Cell Viability Assay

The media was aspired after the 24 h of incubation with the sample products, then adding 20 μL of the neutral red solution (Sigma Aldrich) to each well. The culture was incubated in a humidified chamber at 37 °C for 3 h (5% carbon dioxide). The cells were washed with pre-warmed PBS after incubation, followed by 200 μL of neutral red solubilization solution and left at room temperature for 10 min. A micro plate reader (SpectraMax ^®^ Paradigm^®^ multi-mode detection platform) was used to measure absorbance readings at 540 nm. The cytotoxicity of both the MTT and NR results are reported as a percentage according to the following calculation:

% Cell viability = Sample − Blank/Control − Blank) × 100.(1)

### 3.11. Stability Studies

Environmental stability studies were performed on drug loaded formulations over a period of 12 weeks utilizing selected settings that were intended to simulate everyday use. Generic protocols set by the International Conference on Harmonization (ICH) were considered for the environmental conditions used for this preliminary investigation [71,72]. Samples were kept in airtight, glass jars containing desiccant bags and stored: (a) in a dark enclosure (23 ± 3 °C/65 ± 5% RH), (b) refrigerator (4 ± 2 °C) and (c) under room conditions (24 ± 3 °C/70 ± 5% RH) and tested in triplicate. Formulation weight, disintegration time, drug content uniformity, dissolution pH, inner and outer diameter were selected as indicators for determining the influence of set storage conditions on the physical and chemical stability of these samples. All stability indicators quantified at the end of 12 weeks were compared to measurements conducted at the point when the formulations were freshly prepared (time = 0 weeks).

## 4. Conclusions

Palatable orodispersible film formulations are ideal for patients with swallowing difficulties such as pediatrics because they are stable and dissolve rapidly within the oral cavity in the presence of saliva, without the need to chew or drink water. This current investigation details the successful preparation, optimization and evaluation of an edible, co-polymeric orodispersible pharmaceutical formulation containing pyrazinamide, a model first line antitubercular agent suitable for use in actively infected children. The orodispersible formulation was manufactured by blending polymeric and non-polymeric excipients with drug molecules in an aqueous milieu coupled with the solvent casting approach. The production and optimization processes were facilitated by a one-variable-at-a-time and high performance Box Behnken experimental sesign approaches. The optimized orodispersible formulation was hollow-shaped, uniformly whitish in color, mechanically robust and bendable enough to withstand safe handling. It disintegrated rapidly (34.98 ± 3.00 s) under biorelevant conditions, maintained a close to neutral surrounding pH of 6.90 ± 0.25 and total matrix dissolution and drug release, an indication of complete drug absorption, occurred at approximately 60 min. Drug release from the optimized formulation followed the Korsmeyer–Peppas mathematical model, showing that drug liberation was controlled by anomalous diffusion coupled with matrix disintegration and erosion mechanisms. Pyrazinamide molecules were well incorporated into the formulation matrix and displayed a high loading capacity (25.02 ± 0.71 mg ≡ 101.13 ± 2.03 %*^w^*/*_w_*). According to the WHO, a pediatric patient requires an average dose of 35 mg/kg, meaning that multiple films (relative to body weight) may be needed per child; an approach not unusual in TB management with oral or water dispersible tablets. This may therefore be more usable in under 5-year-old children and, should not pose any choking hazards considering the rapidly disintegrating characteristic of the fabricated pyrazinamide films which does not necessitate the use of water for swallowing. Captured SEM micrographs and digital photographs showed that the drug formulation matrix was micro-structured and also confirmed its quick disintegration sequence. The orodispersible drug preparation was thermodynamically and environmentally stable under specific storage conditions based on findings from physicochemical characterization (TGA, DSC, FTIR, XRD, BET analyses) and stability testing processes. Preliminary organoleptic and cell toxicity enquiries presented the drug formulation as palatable, easy-to-handle and biocompatible under applied test conditions. In conclusion, the orodispersible pharmaceutical formulation developed herein can potentially ease some of the current global challenges associated with the safe administration of TB antibiotics in pediatric patients to aid desirable pharmacotherapeutic outcome. Besides, the carrier matrix designed in this study may be used as is or even modified to accommodate and safely improve the release/absorption of other antitubercular agents for use in children.

## Figures and Tables

**Table 1:** Response parameters generated based on the quadratic experimental design template.

Formulation	Weight (mg)	Disintegration Time (minutes)	Dissolution pH
F1	91.66 ± 1.55	2.57 ± 0.34	7.33 ± 0.02
F2	65.60 ± 2.58	0.47 ± 0.15	6.69 ± 0.04
F3	106.80 ± 2.00	1.59 ± 0.50	7.04 ± 0.02
F4	100.00 ± 2.38	1.24 ± 0.23	6.96 ± 0.05
F5	105.83 ± 3.95	1.72 ± 0.42	6.78 ± 0.04
F6*	87.86 ± 2.11	1.50 ± 0.09	6.59 ± 0.23
F7	75.07 ± 4.45	1.19 ± 0.06	6.94 ± 0.02
F8	60.87 ± 3.80	0.44 ± 0.04	6.96 ± 0.03
F9*	86.50 ± 2.59	1.45 ± 0.24	6.83 ± 0.06
F10	81.23 ± 1.64	3.46 ±0.51	6.98 ± 0.01
F11	81.50 ± 1.64	5.40 ± 0.10	7.30 ± 0.03
F12	84.77 ± 6.96	1.94 ± 0.62	7.06 ± 0.03
F13	112.06 ± 18.98	3.62 ±2.90	7.23 ± 0.14
F14	121.4 ± 8.00	5.44 ± 0.14	7.01 ± 0.06
F15	101.33 ± 4.51	4.84 ± 0.37	6.70 ± 0.06
F16	82.63 ± 0.06	4.20 ± 0.13	7.29 ± 0.03
F17	82.07 ± 3.04	5.03 ± 1.02	7.07 ± 0.03
F18	73.67 ± 3.16	4.83 ± 1.54	7.05 ± 0.02
F19	77.3 ± 1.42	4.02 ± 0.58	7.02 ± 0.06
F20	77.3 ± 14.03	2.58 ± 0.53	6.95 ± 0.06
F21	109.47 ± 2.39	2.57 ± 1.28	7.33 ± 0.41
F22	98.23 ± 15.06	2.73 ± 1.54	7.00 ± 0.05
F23*	90.30 ± 1.34	1.08 ± 0.10	7.10 ± 0.01
F24	80.97 ± 1.23	1.67 ± 0.57	7.43 ± 0.01
F25	114.97 ± 10.08	5.67 ± 0.42	7.05 ± 0.04
F26	78.40 ± 5.56	0.20 ± 0.09	7.02 ± 0.05
F27	70.37 ± 3.75	0.72 ± 0.34	7.41 ± 0.06

* represents the centrepoint experimental runs.

**Table 2:** Optimized formula and model summary of fitted and experimental outputs as it relates to the experimental design template.

Optimized Formula	Validation of Predicted Outputs with Experimental Values (*n* = 3)			
***Factor Levels***	***Responses***	***Desirability Level***	***Predicted***	***Observed***
**X_1_** = 1.000 g	**Y_1_**	0.980	0.620 min	0.583 ± 0.050 min
**X_2_** = 0.483 g	**Y_2_**	1.000	7.000	6.900 ± 0.250
**X_3_** = 0.058 g	**Y_3_**	0.994	65.333 mg	57.500 ± 0.002 mg
**X_4_** = 0.539 g				

**Note**: **X_1_** = Polyvinyl alcohol polyethylene glycol (Kollicoat^®^ IR); **X_2_** = Sodium starch glycolate (Primojel^®^); **X_3_** = Citric acid; **X_4_** = Xylitol, **Y_1_** = Disintegration time; **Y_2_** = Disintegration pH; **Y_3_** = Formulation weight.

**Table 3:** Stability indicators recorded under different storage conditions.

Stability Indicators	0 Weeks	Varying Storage Conditions at 12 Weeks		
		I *	II *	III *
Mass (mg)	57.500 ± 0.002	56.100 ± 0.472	58.600 ± 0.321	58.530 ± 1.531
Outer diameter (mm)	11.000 ± 0.426	11.000 ± 0.577	11.000 ± 0.577	11.667 ± 0.577
Inner diameter (mm)	10.000 ± 0.520	10.000 ± 0.577	10.000 ± 0.577	10.667 ± 0.577
Disintegration time (min)	0.583 ± 0.050	0.533 ± 2.510	0.467 ± 3.050	0.483 ± 1.154
Dissolution pH	6.900 ± 0.250	7.290 ± 0.0.015	7.640 ± 0.071	7.633 ± 0.041
% drug content	101.000 ± 2.030	88.000 ± 15.530	84.000 ± 3.020	33.333 ± 10.408
Colour change	None	None	None	Yes

* Note: **I**—refrigerator (4 ± 2 °C); **II**—dark enclosure (23 ± 3 °C/65 ± 5% RH); **III**—regular room conditions (24 ± 3 °C/70 ± 5% RH).

**Table 4:** Independent variables employed for the Box Behnken design template.

Independent Variables	Levels		
	−1	0	+1
**X_1_**: Polyvinyl alcohol polyethylene glycol (g)	1.000	2.000	3.000
**X_2_**: Sodium starch glycolate (g)	0.000	0.500	1.000
**X_3_**: Citric acid (g)	0.025	0.063	0.100
**X_4_**: Xylitol (g)	0.300	0.650	1.000

**Table 5:** Box Behnken design template.

Formulation	X_1_	X_2_	X_3_	X_4_
F1	3.0000	0.5000	0.0250	0.6500
F2	2.0000	1.0000	0.0625	1.0000
F3	3.0000	1.0000	0.0625	0.6500
F4	1.0000	0.5000	0.1000	0.6500
F5	3.0000	0.5000	0.1000	0.6500
F6 *	2.0000	0.5000	0.0625	0.6500
F7	1.0000	1.0000	0.0625	0.6500
F8	1.0000	0.5000	0.0625	0.3000
F9 *	2.0000	0.5000	0.0625	0.6500
F10	2.0000	0.5000	0.1000	0.3000
F11	2.0000	0.0000	0.0250	0.6500
F12	2.0000	1.0000	0.0625	0.3000
F13	2.0000	0.5000	0.0250	1.0000
F14	1.0000	0.0000	0.0625	0.6500
F15	3.0000	0.5000	0.0625	0.3000
F16	2.0000	1.0000	0.0250	0.6500
F17	2.0000	0.0000	0.0625	1.0000
F18	2.0000	0.0000	0.0625	0.3000
F19	2.0000	0.0000	0.1000	0.6500
F20	2.0000	0.5000	0.1000	1.0000
F21	3.0000	0.5000	0.0625	1.0000
F22	3.0000	0.0000	0.0625	0.6500
F23 *	2.0000	0.5000	0.0625	0.6500
F24	2.0000	0.5000	0.0250	0.3000
F25	2.0000	1.0000	0.1000	0.6500
F26	1.0000	0.5000	0.0625	1.0000
F27	1.0000	0.5000	0.0250	0.6500

**Note**: * indicates the experimental design center points; **X_1_** = Polyvinyl alcohol polyethylene glycol (Kollicoat^®^ IR); **X_2_** = Sodium starch glycolate; **X_3_** = Citric acid; **X_4_** = Xylitol. Each orodispersible film variant blend (i.e., F1–F27) contained 20 mL deionized water as solvent, 500 mg pyrazinamide as model drug and produced an average of 20 orodispersible film formulations per variant blend meaning that a single film formulation was loaded with approximately 25 mg pyrazinamide.

**Table 6:** Model summary of optimization constrains and statistical significance of the selected response parameters.

Dependent Variables	Constrain	Lower	Upper	Goal	R	*p*-Value
**Y_1_**: Disintegration time (minutes)	Minimize	0.500	0.700	0.600	0.910	0.002
**Y_2_**: Disintegration pH	Target	6.900	7.100	7.000	0.940	0.001
**Y_3_**: Formulation weight (mg)	Minimize	50.00	70.00	60.00	0.901	0.020
